# Comparison of pulmonary function changes between patients receiving neoadjuvant chemotherapy and chemoradiotherapy prior to minimally invasive esophagectomy: a randomized and controlled trial

**DOI:** 10.1007/s00423-022-02646-x

**Published:** 2022-08-25

**Authors:** Xiaosang Chen, Mingjun Du, Han Tang, Hao Wang, Yong Fang, Miao Lin, Jun Yin, Lijie Tan, Yaxing Shen

**Affiliations:** 1 Department of Thoracic Surgery, Zhongshan Hospital, Fudan University, Shanghai, 200032 China; 2 Department of Thoracic Surgery, National Cancer Center/National Clinical Research Center for Cancer/Cancer Hospital, Chinese Academy of Medical Sciences and Peking Union Medical College, Beijing, 10021 China

**Keywords:** Esophageal cancer, Neo-adjuvant therapy, Pulmonary function

## Abstract

**Purpose:**

Adequate pulmonary function is important for patients undergoing surgical resection of esophageal cancer, especially those that received neoadjuvant therapy. However, it is unknown if pre-operative radiation affects pulmonary function differently compared to chemotherapy. The purpose of this study was to compare changes in pulmonary function between patients undergoing minimally invasive esophagectomy (MIE) who received neoadjuvant chemotherapy or chemoradiotherapy.

**Methods:**

Between March 2017 and March 2018, esophageal cancer patients requiring neoadjuvant therapy were prospectively enrolled and randomly assigned to receive chemotherapy (CT) or chemoradiotherapy (CRT) before MIE. All patients received pulmonary function testing before and after the neoadjuvant therapy. Changes in pulmonary function, operative data, and pulmonary complications were compared between the 2 groups.

**Results:**

A total of 71 patients were randomized and underwent MIE after receiving CT (*n* = 34) or CRT (*n* = 37). Baseline clinical characteristics were comparable between the 2 groups. The CRT group experienced a greater decrease of forced expiratory volume at 1 s (FEV_1_) (2.66 to 2.18 L, *p* = 0.023) and diffusion capacity of the lung for carbon monoxide divided by the mean alveolar volume (DLCO/Va) (17.3%, *p* < 0.001) than the CT group (FEV_1_ 2.53 to 2.41 L; DLCO/Va 4.8%). The incidence of pulmonary complications was higher in the CRT group (13.51 vs. 8.82%), but the difference was not significant (*p* = 0.532).

**Conclusions:**

Preoperative CRT affects pulmonary function more than CT alone, but does not increase the risk of pulmonary complications in patients undergoing MIE.

**Supplementary Information:**

The online version contains supplementary material available at 10.1007/s00423-022-02646-x.

## Introduction

Neoadjuvant therapy is indicated in the treatment of locally advanced esophageal cancer due to both surgical and oncological benefits [1–5]. However, neoadjuvant therapy can increase the risk of pulmonary complications, and thus peri-operative morbidity and mortality [6, 7]. As such, adequate pulmonary function is important for achieving good outcomes in patients undergoing surgical resection of esophageal cancer.

Studies have provided interesting results with respect to the relation between pulmonary function and postoperative morbidity. Cerfolio et al. [8] described a decrease of pulmonary function after neoadjuvant therapy, which predicted increased postoperative morbidity in patients with non-small cell lung cancer (NSCLC) undergoing surgery. Abou-Jawde et al. [9] reported that neoadjuvant therapy was associated with decreased pulmonary function and increased acute respiratory complications in patients with esophageal cancer undergoing surgery. However, their results were from a retrospective analysis of 3 clinical trials with high heterogeneity. Currently, it is not known if neoadjuvant radiotherapy affects pulmonary function differently than chemotherapy alone, and if the changes in pulmonary function result in a different rate of surgical pulmonary complications.

Based on our previous experience performing minimally invasive esophagectomy (MIE) [10, 11], the purpose of this study was to compare changes in pulmonary function between patients undergoing MIE who received neoadjuvant chemotherapy or chemoradiotherapy, and determine if there were differences in outcomes and complications between the 2 groups.

## Patients and methods

### Patients

This study was a prospective, randomized, and controlled trial, and was registered at Clinicaltrials.gov (NCT03001596) [12]. It was approved by the Ethics Committee of Zhongshan Hospital, Fudan University, China (B2016-177R). Consecutive patients with locally advanced esophageal cancer, pursuant a standard staging procedure (endoscopy, tissue biopsy, computed tomography [CT], and positron emission tomography [PET]), and a histologically proven diagnosis of locally advanced esophageal cancer (cT_3-4a_N_0-1_M_0_) were enrolled. Complete inclusion and exclusion criteria are summarized in Supplementary Table 1.

Patients were randomized to receive neoadjuvant chemotherapy (CT) or neoadjuvant chemoradiotherapy (CRT) using a computer-generated list. Patients were randomly assigned to either group using sequentially numbered sealed envelopes containing information that disclosed the type of treatment.

### Neoadjuvant therapy protocols

#### Neoadjuvant CT

Neoadjuvant CT consisted of 2 cycles of paclitaxel 175 mg/m^2^ on day 1 and cisplatin 75 mg/m^2^ on day 1/2 by intravenous infusion, with 3 weeks between cycles.

#### Neoadjuvant CRT

Neoadjuvant CRT consisted of concurrent preoperative radiotherapy and chemotherapy.

Radiation was administered based on the volume and location of the tumor. CT-based planning was performed for each patient. The total dose was 40 Gy, given in daily single 2 Gy fractions on days 1–5, 8–12, 15–19, and 22–26. Chemotherapy consisting of paclitaxel 50 mg/m^2^ and cisplatin 25 mg/m^2^ was administered on day 6, 13, 20, and 27.

### Surgery following neoadjuvant therapy

MIE was performed 4–8 weeks after completion of neoadjuvant therapy. MIE consisted of 3 stages (thoracic, abdominal, and cervical stages), and the details of the surgery have been previously published [10, 11]. The thoracic stage included esophageal mobilization and mediastinal lymphadenectomy. Patients were placed in a semi-prone position with the right arm raised above the head and the right side of the operating table slightly raised. The surgeon stood on the right side of the patient. An observation port was placed at the seventh intercostal space (ICS) along the mid-axillary line, and another 10-mm port was placed at the ninth ICS along the scapular line. Two 5-mm ports were placed at the third ICS along the mid-axillary line, and just inferior to the tip of the scapula, respectively. An artificial CO_2_ pneumothorax was achieved at a pressure of 8 mm Hg. After thoracoscopic exploration, the azygous vein was double-ligated by Hem-o-locks and then divided, followed by mobilization of the thoracic esophagus. The thoracic duct was identified and carefully preserved. The esophageal arteries were divided with a harmonic scalpel, and mediastinal lymph nodes, including lymph nodes along the bilateral recurrent laryngeal nerves and subcarinal lymph node, were removed en bloc. The thoracic procedure was completed by placement of intercostal drains and closure of the thoracic ports. The abdominal and cervical stages were the same as described previously [13–16]. The operation concluded with closure of the cervical and abdominal incisions in layers.

### Pulmonary function testing

Pulmonary function tests (PFTs) were performed before and 6 weeks after neoadjuvant therapy, using the Spirometer System (Biomedin, Padua, Italy). PFT parameters collected included vital capacity (VC), forced vital capacity (FVC), forced expiratory volume at 1 s (FEV_1_), total lung capacity (TLC), residual volume (RV), peak expiratory flow (PEF), diffusion capacity of the lung for carbon monoxide by single breath method (DLCO/SB), and the DLCO divided by the mean alveolar volume (DLCO/Va). Blood atrial gas analysis was performed by sampling of the radial artery blood.

### Pulmonary complications

Postoperative complications were defined by reference to the Society of Thoracic Surgeons (STS) database, and definitions from the official website of the International Society for the Diseases of Esophagus (ISDE). Chest radiographs were obtained to evaluate for possible pulmonary complications when necessary. Pulmonary complications were defined as the primary morbidity in the following situations: (1) a therapeutic bronchoscopy/tracheotomy was performed due to bronchial secretions; (2) pneumonia was diagnosed per clinical and radiographic criteria; (3) the occurrence of acute lung injury/acute respiratory distress syndrome (ARDS); (4) the development of a pleural effusion requiring an additional drainage procedure; (5) mucous plugging requiring bronchoscopy; and (6) occurrence of a pulmonary embolism. Mortality was defined as death before discharge or within 30 days of the operation.

### Statistical analysis

Clinical data for all enrolled patients were collected from the clinical database of our institution. All data were tabulated using Microsoft Excel (Microsoft, Redmond, WA), and statistical analysis was performed with SPSS version 17.0 software (SPSS, Inc., Chicago, IL). Variables were compared using the Mann–Whitney test, the Student *t* test, the Chi-square test, and Fisher’s exact test, as appropriate. A 2-tailed value of *p* < 0.05 was considered statistically significant.

## Results

### Patient demographic characteristics

Recruitment occurred from March 2017 to March 2018, and a total of 76 patients met the inclusion criteria and were deemed eligible for the study. One patient of the 76 declined to participate in the study and the remaining 75 patients were randomized to the CT group or CRT group. Subsequently, 4 patients discontinued the study (2 with disease progression and 2 who subsequently refused surgery). Thus, 71 patients were included in the analysis and underwent MIE after receiving CT (*n* = 34) or CRT (*n* = 37).

**Fig. 1:**
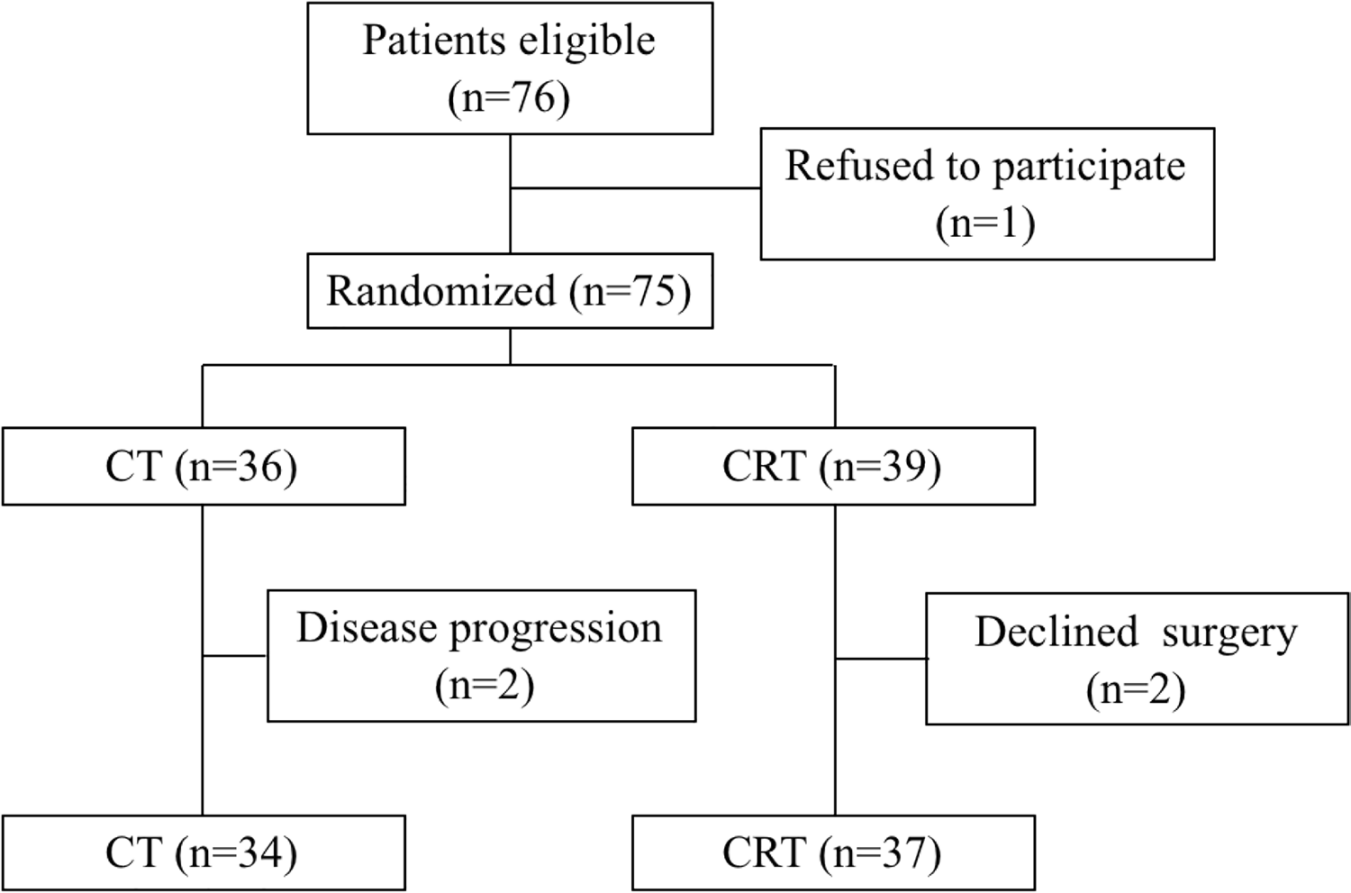
Flow diagram of patient inclusion. CT, chemotherapy; CRT, chemoradiotherapy

A flow diagram of patient inclusion is shown in Fig. 1, and patient demographic and clinical features are summarized in Table 1. Specifically, no patients with asthma, chronic obstructive pulmonary disease, or interstitial lung disease were enrolled. No esophageal tumor initially invaded or made an impression of the trachea were found from CT-scan in this study.

**Table 1:** Patient demographic and clinical characteristics

	CT (*n* = 34)	CRT (*n* = 37)	*p* value
Age (years)	62.7 ± 6.5	64.3 ± 7.1	0.779*
Sex (M:F)	26:8	29:8	0.848†
Location (U:M:L)	5:25:4	6:23:8	0.974‡
cStage (T3:T4)	27:7	32:5	0.427‡
Ex-smokers	14	15	0.957†
ASA (I:II)	12:22	14:23	0.824†

Data are presented as mean ± standard deviation, or number

*CT*, chemotherapy; *CRT*, chemo-radiation therapy; *ASA*, American Society of Anesthesiologists

^*^Student *t* test

^†^Chi-square test

^‡^Fisher exact test

### Changes in pulmonary function

**Fig. 2:**
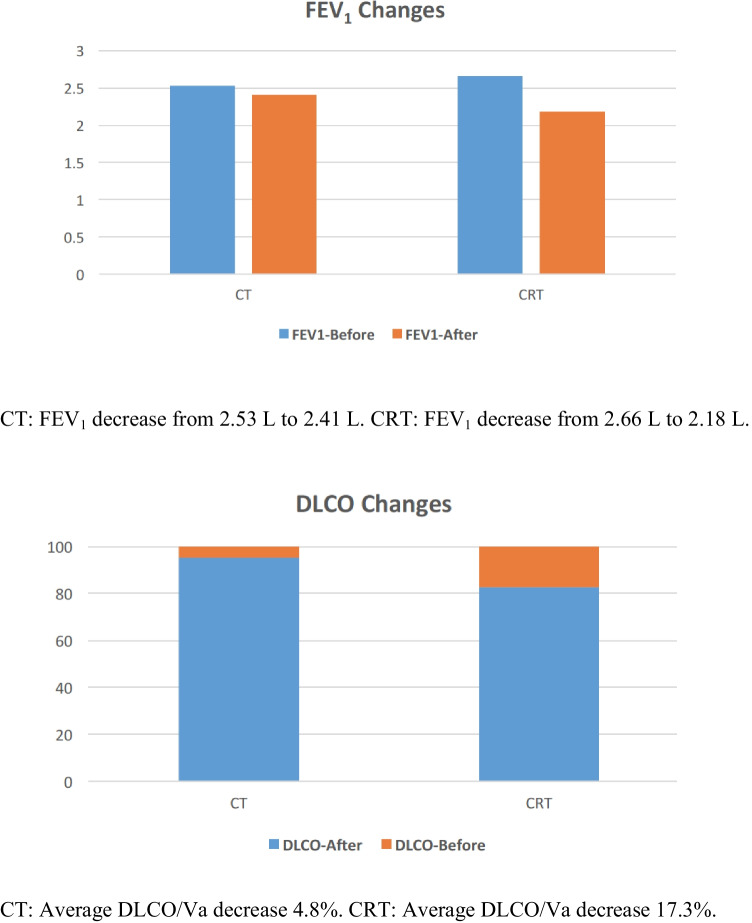
Changes in pulmonary function before and after neoadjuvant therapy. CT: FEV_1_ decrease from 2.53 to 2.41 L. CRT: FEV_1_ decrease from 2.66 to 2.18 L. CT: Average DLCO/Va decrease 4.8%. CRT: Average DLCO/Va decrease 17.3%. The CRT group experienced a greater decrease in both FEV_1_ (*p* = 0.023) and DLCO/Va (*p* < 0.001) than the CT group. CT, chemotherapy; CRT, chemoradiotherapy

All patients received PFTs before and 6 weeks after the neoadjuvant therapy during pre-operation assessment, and no patients were excluded from the surgery due to decreased pulmonary function. In the CRT group, the average FEV_1_ decreased from 2.66 to 2.18 L, while in the CT group the average FEV_1_ decreased from 2.53 to 2.41 L. The decrease of FEV_1_ in the CRT group reached statistical difference (*p* = 0.023), while the decrease in the CT group did not. The DLCO/Va decreased by 17.3% in the CRT group and 4.8% in CT group (*p* < 0.001). A summary of PFTs results is shown in Fig. 2.

### Pulmonary complications

**Fig. 3:**
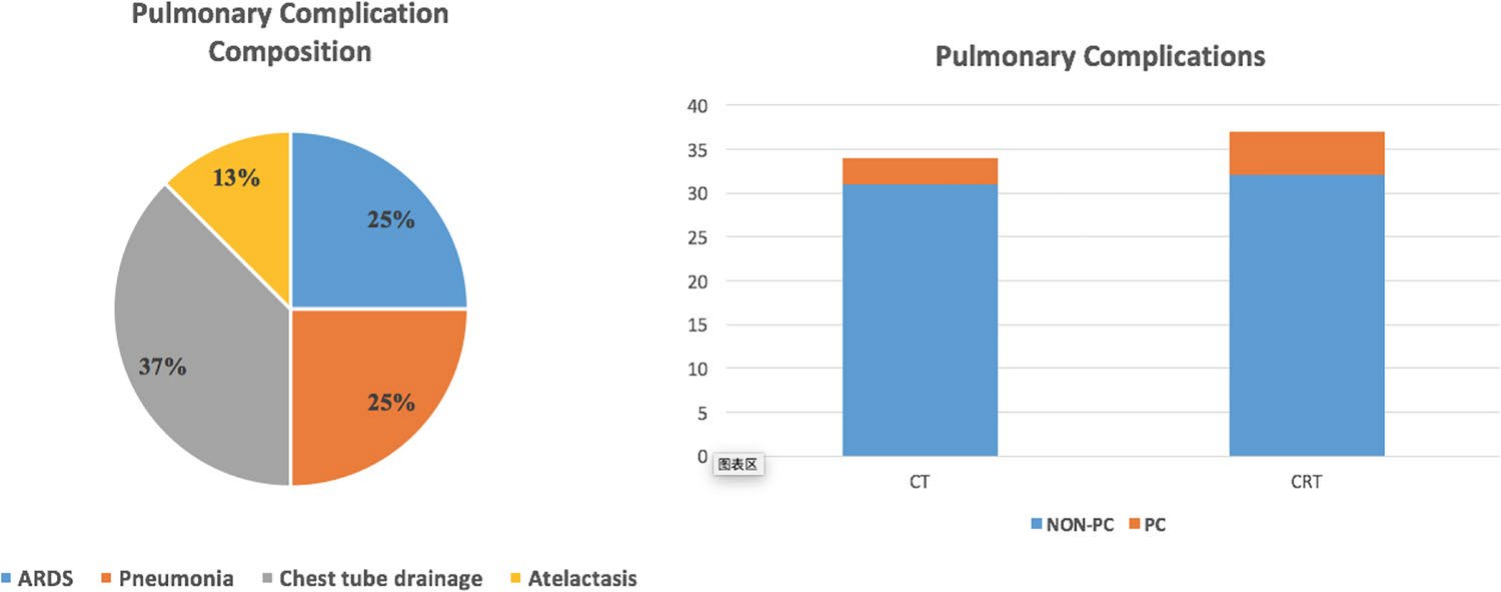
Pulmonary complications after minimally invasive esophagectomy. A total of 8 patients (11.27%) developed pulmonary complications: 5 patients (13.51%) in CRT group and 3 patients (8.82%) in CT group. The frequency of pulmonary complications between the 2 groups was not statistically different (*p* = 0.532). Pulmonary complications included 2 cases of ARDS, 2 cases of pneumonia, 3 cases of pleural effusion requiring chest tube insertion and drainage, and 1 case of atelectasis. All complications resolved with appropriate management

A total of 8 patients (11.27%) developed pulmonary complications: 5 patients (13.51%) in CRT group and 3 patients (8.82%) in CT group. The frequency of pulmonary complications between the 2 groups was not statistically different (*p* = 0.532). Pulmonary complications included 2 cases of ARDS, 2 cases of pneumonia, 3 cases of pleural effusion requiring chest tube insertion and drainage, and 1 case of atelectasis. All complications resolved with appropriate management. In the 2 cases of ARDS, patients were provided nasal/mask oxygen inhalation. The other 6 patients were provided nasal oxygen inhalation intermittently when they felt dyspnea. A summary of pulmonary complications is shown in Fig. 3.

## Discussion

The results of this study showed that patients who received CRT had a greater decrease in FEV_1_ and DLCO/Va than patients that received CT; however, the frequency of pulmonary complications was not different between the 2 groups. How far these PFT parameters decline correlates with clinical worsening of the patients, and remains unclear at this current study. Minimally clinical important difference (MCID) is a useful expression defined as the minimal amount of change required to be confident that a patient has truly changed. Considering the fact that there has been little evidence on MCID for FEV_1_ and DLCO in patients underwent neoadjuvant therapy and the relatively small sample size of this study, MCID has not been evaluated in this study. MCID would be estimated in further work with a bigger sample size to determine if changes of PFTs are clinically important for the patient.

Published evidence has shown that CRT or CT provides a survival benefit over surgery alone for locally advanced esophageal cancer [5, 6]. However, neoadjuvant therapy potentially increases the risk of postoperative morbidity and mortality and thus imposes restrictions on the application of neoadjuvant therapy, especially CRT. Recently, Yong et al. [17] conducted a randomized trial comparing the outcomes of CRT followed by surgery with surgery alone in esophageal cancer patients. The study showed that 17% of patients in the CRT group did not undergo surgery after CRT, and 54.3% of CRT patients developed grade 3 or 4 hematologic toxicity, suggesting that neoadjuvant therapy increased perioperative risks. MIE is associated with less trauma than open surgery and a similar curative effect, and MIE has been shown to be associated with improved perioperative outcomes, including a decrease of pulmonary complications [18, 19]. However, respiratory morbidity is still the most frequent complication after esophagectomy despite the development of advanced surgical and perioperative management techniques.

Many studies have demonstrated the detrimental effects of neoadjuvant therapy on pulmonary function [20, 21]. A correlation between PFTs changes after induction therapy and respiratory morbidity has been reported in patients with non-small cell lung cancer (NSCLC). Cerfolio et al. [8] reported that a decrease of DLCO/Va after neoadjuvant therapy may predict increased postoperative morbidity, especially if the decrease is 8% or greater. Margaritora et al. [22] reported a 22.8% decrease of DLCO in patients after induction chemoradiotherapy, but they did not compare the relations between the change of PFTs and incidence of postoperative complications. Despite increasing application of neoadjuvant therapy, its impact on pulmonary function and pulmonary complications has not been fully investigated in the setting of esophageal cancer surgery. Abou-Jawde et al. [9] reported that CRT was associated with significant decreased DLCO in patients with esophageal cancer, and the decrease was greater in patients that received 45 Gy than those that received 30 Gy. Post-CRT DLCO was also worse in patients with postoperative acute respiratory complications. However, the study was a retrospective analysis of 3 clinical trials, and some of PFT data, particularly pretreatment DLCO values, were missing. Considering the retrospective nature and heterogeneity of previous studies, we conducted this prospective, randomized, controlled trial to evaluate the relations between PFT changes and postoperative pulmonary complications in patients undergoing MIE, and to determine if there were differences between patients that received neoadjuvant CT or CRT.

A prior study reported that changes in pulmonary function were an independent risk factor for primary pulmonary complications in patients undergoing surgery for esophageal cancer [23]. Pulmonary complications were defined as a primary postoperative morbidity in this study because secondary pulmonary complications are more serious conditions, such as aspiration and leakage after surgery [24]. Since lymphadenectomy along the bilateral recurrent laryngeal nerves is conventionally performed during esophagectomy, patients are at high risk of vocal cord palsy and aspiration pneumonia. In addition, nasogastric tubes are commonly used for gastric conduit decompression after esophagectomy, which can cause discomfort and inhibit expectoration, and thus increase the risk of aspiration [25]. Anastomotic leakage, which occurs with relatively high frequency after esophagectomy, can lead to severe infection in the thoracic cavity. Additionally, a patient’s nutritional and immune state can be severely affected, causing rapid deterioration of their general condition which predisposes to serious infection. Compared to chemotherapy alone, neoadjuvant radiation increases tissue edema, inflammation, and fibrosis, with a subsequent higher risk of perioperative morbidity. Hence, secondary pulmonary complications tend to be the consequence of complicated surgical procedure and other postoperative morbidities, rather than a patient’s respiratory reserve, and thus were excluded from the evaluation in this study.

It has been reported that spirometry can help predict the likelihood of pulmonary complications. Generally, FEV_1_ reflects pulmonary ventilation function, and DLCO represents functional gas exchange capacity [26]. Takeda et al. [27] and Leo et al. [6] reported lower DLCO/Va and higher FEV_1_ after induction therapy in patients with NSCLC. Multivariate analysis revealed that DLCO was an independent factor predictive of pulmonary morbidity. Improved FEV_1_ after neoadjuvant therapy may result from relief in bronchial obstruction caused by tumor extension or enlarged lymph nodes. Unlike patients with NSCLC whose volume of lung may improve, patients with esophageal cancer tend to have unchanged or worse FEV_1_ after neoadjuvant therapy. Ferguson et al. [23] reported FEV_1_ can help predict the likelihood of pulmonary complications after esophagectomy. Radiation and chemotherapy can worsen gas exchange, and it has been proposed that DLCO assessed after neoadjuvant therapy was superior to FEV_1_ as a predictor of pulmonary complications [22].

Some chemotherapeutic agents, such as cisplatin, can cause a decline in alveolo-capillary membrane diffusion capacity by a mechanism that resembles the pathogenesis of ARDS [28, 29], and the addition of radiation to chemotherapy can enhance pulmonary toxicity. Since the degree of subclinical damage to the alveolo-capillary membrane is proportional to DLCO/Va decrease, the measurement of DLCO/Va may be more sensitive for predicting pulmonary complications.

No patients died in our study. Though patients in the CRT group had a greater decrease of FEV_1_ and DLCO/Va than patients in the CT group, the frequency of pulmonary complications was similar between the 2 groups. It is possible that the lack of difference in the pulmonary complication rate was due to the relatively small sample size. However, our results do provide proof that CRT depresses pulmonary function more than CT alone. Possible explanations include that radiation-induced pulmonary fibrosis and lymphocytopenia may occur as a result of radiation, resulting in interstitial infiltrates. In this study, 2 cases of ARDS and 2 cases of pneumonia (3 patients in CRT group and 1 patient in CT group) were found in CT-scan, which could be the result of interstitial infiltrates. A higher rate of interstitial infiltrates in CT-scan was found in CRT group, correlated to the decline of FEV_1_ and DLCO. The correlation between pulmonary complications and decline of PFT parameters remains unclear, which should be explored in some pulmonary function rehabilitation study following this observational research. One important clinical finding of this study is that if DLCO/Va and FEV_1_ decrease significantly after neoadjuvant therapy, the risk of pulmonary complications can be increased. Our results suggest that evaluation of PFTs may help identify patients at increased risk of pulmonary complications and thus permit appropriate interventions to decrease the risk and improve outcomes.

The limitations of this study are that it was performed at a single institution, and the number of enrolled patients was limited. The strengths of this study include the well-defined inclusion criteria and the homogeneous patient population.

## Conclusions

For esophageal cancer patients undergoing neoadjuvant therapy, CRT affects pulmonary function more than CT alone, and may increase the risk of pulmonary complications. A decline of FEV_1_ and DLCO/Va after neoadjuvant therapy should be considered in the preoperative risk assessment of patients undergoing MIE.

## Supplementary Information

Below is the link to the electronic supplementary material.

Supplementary file1 (DOCX 17 KB)
