# Supplementary material for: Comparison of pulmonary function changes between patients receiving neoadjuvant chemotherapy and chemoradiotherapy prior to minimally invasive esophagectomy: a randomized and controlled trial
Source: Langenbecks Arch Surg. 2022 Aug 25;407(7):2673–80. doi: 10.1007/s00423-022-02646-x (PMC9640419; doi:10.1007/s00423-022-02646-x)
Supplement: Supplementary file 1 — Supplementary file1 (DOCX 17 KB) [file 423_2022_2646_MOESM1_ESM.docx]

**Supplementary Table 1. Inclusion and exclusion criteria**

**Inclusion criteria**

1. Age between 18 years and 75 years
2. Histologically-confirmed squamous cell carcinoma of the esophagus
3. Tumor of the esophagus located in the thoracic cavity;
4. Pre-treatment stage cT_3-4a_N_0-1_M_0_ (AJCC/UICC 7th Edition)
5. Eastern Cooperative Oncology Group (ECOG) performance status 0-1
6. Adequate respiratory function with FEV_1_ ≥ 1.2 L, FEV_1_% ≥ 50%, and DLCO/Va ≥ 50% as found in pulmonary function tests
7. Adequate cardiac function with left ventricular ejection fraction > 50%
8. Adequate bone marrow function (white blood cell count > 4 × 10^9^/L; Neutrophils > 2.0 × 10^9^/L; hemoglobin > 90 g/L; platelets > 100 × 10^9^ /L)
9. Adequate liver function (total bilirubin < 1.5× upper level of normal (ULN); aspartate transaminase (AST) and alanine transaminase (ALT) < 1.5× ULN)
10. Adequate renal function (glomerular filtration rate > 60 ml/min; serum creatinine ≤ 120 μmol/L)
11. Provided written informed consent for the study

**Exclusion criteria**

1. Non-squamous cell carcinoma histology
2. Advanced inoperable or metastatic esophageal cancer
3. Pre-treatment stage cT_1-2_N_0-1_M_0_ (AJCC/UICC 7th Edition)
4. Pre-treatment stage cN_2–3_ or cT_4b_ (non-curatively resectable verified by the local surgical investigator, AJCC/UICC 7th Edition)
5. Another previous or current malignant disease which is likely to interfere with treatment or the assessment of response in the judgement of the local surgical investigator
6. A significant medical condition which may result in inability to tolerate surgery (e.g., symptomatic coronary artery disease or myocardial infarction within last 12 months, clinically significant lung disease, clinically significant bone marrow, liver, or renal function disorder)
7. Pregnant or lactating women and fertile women who will not be using contraception during the trial
8. Allergic to the chemotherapy regimens
9. Participation in another intervention clinical trial with interference to the chemotherapeutic or chemoradio therapeutic intervention during this study or during the last 30 days prior to informed consent
10. Expected lack of compliance with the protocol
